# Demographic and Clinical Features of Pediatric Bell’s Palsy: A 26-Year Experience at a Tertiary Care Hospital in Riyadh

**DOI:** 10.7759/cureus.98360

**Published:** 2025-12-03

**Authors:** Waleed Altwajiri, Abdulelah Alshamrani, Abdulaziz Almughamis, Abdulaziz Alnufaei, Abdullah Alkharboosh, Salman Alotaibi

**Affiliations:** 1 Pediatric Neurology, King Abdulaziz Medical City, Riyadh, SAU; 2 Medicine, King Saud Bin Abdulaziz University for Health Sciences College of Medicine, Riyadh, SAU

**Keywords:** bell's palsy, facial deviation, facial nerve paralysis, pediatrics, seventh cranial nerve

## Abstract

Background: Bell’s palsy is the most common peripheral paralysis of the seventh cranial nerve, characterized by a rapid and unilateral onset. The condition is encountered across different regions and age groups, with its frequency varying by population.

Aim of the study: This study aimed to identify the detailed demographics and evaluate the duration of illness, associated symptoms, complications, and recurrence rates among 301 children diagnosed with Bell’s palsy at King Abdulaziz Medical City (KAMC) in Riyadh, Saudi Arabia. Additionally, we aimed to examine different treatment modalities and compare prognostic factors. This study sought to address existing knowledge gaps regarding the clinical features and complications of Bell’s palsy in pediatric patients.

Methods: In this retrospective study, data were collected from children aged 0-14 years who had been diagnosed with Bell’s palsy. All patients meeting the inclusion criteria at KAMC between 1997 and 2023 were included.

Results: Facial weakness was observed in 112 (37%) patients, while alterations in the facial/mouth/nasal angle were noted in 206 (68%). Facial numbness was absent in 288 (96%) cases. Regarding complications, 281 (94%) had none, two (0.67%) did not achieve full recovery, one (0.33%) experienced a prolonged disease course, 12 (4%) reported persistent abnormal facial sensations, and two (0.67%) had vision-related issues. Bilateral Bell’s palsy occurred in only one patient (0.33%). Prednisolone therapy was administered to 243 (81%) patients, and eye care measures were provided in 216 (72%) cases.

Conclusion: Bell’s palsy in children generally carries a favorable prognosis, with most cases resolving fully without complications. Facial deviation and difficulty closing the eye are common clinical manifestations. Oral corticosteroids represent the most frequently employed treatment, though evidence for their efficacy remains mixed. In our study, approximately 20% of patients experienced recurrence, and some may have multiple episodes. Further research is needed to identify optimal treatment strategies and to clarify prognostic factors.

## Introduction

Bell’s palsy, first described by the anatomist Charles Bell, is an idiopathic peripheral facial nerve (cranial nerve VII) condition that manifests as a sudden, unilateral paralysis of the face [1]. Commonly referred to as acute idiopathic peripheral facial palsy, Bell’s palsy affects individuals worldwide, with annual incidence rates ranging from 11.5 to 53.3 cases per 100,000 people in different populations [2]. In addition, a local retrospective study conducted at King Abdulaziz Medical City (KAMC), Riyadh, Saudi Arabia, between 1994 and 2005 found that among 83,067 patients, 29 infants were diagnosed with traumatic facial palsy [3].

Bell’s palsy presents with a variety of clinical features and can lead to both medical and non-medical complications. The most frequently observed sign is a facial droop and difficulty making facial expressions [4]. Additional features noted at onset include retroauricular pain, dry eye, dysgeusia, hyperlacrimation, and aural fullness [4,5,6]. Other manifestations encompass postauricular pain, dry eye, drooling, loss of taste, increased sensitivity to sound and pain, and xerostomia [4,5,6]. Xerostomia, in particular, is associated with a poor prognosis [4]. Persistent Bell's Palsy may result in facial muscle atrophy if present for extended periods, and severe cases often lead to dry eyes due to incomplete eyelid closure [4].

While facial muscle paralysis may arise from various etiologies, such as traumatic, congenital, viral, and neoplastic factors, Bell’s palsy is considered the most common cause of facial paralysis in children [7]. Pathophysiological mechanisms include inflammation, edema, lymphocytic infiltration, demyelination, or axonal degeneration of the facial nerve fibers [8]. Pediatric Bell’s palsy typically follows a benign course; most children show improvement within two weeks, and the majority regain normal facial function by six months [1]. Up to 90% of children under the age of 14 experience spontaneous recovery [9]. A Turkish study examining prognostic factors in pediatric Bell’s palsy reported that 93.6% of children demonstrated complete or nearly complete recovery, while the remaining cases showed partial improvement [10].

Treatment strategies for Bell’s palsy often involve the use of corticosteroids. Patients may receive oral or intravenous methylprednisolone (1 mg/kg/day) tapered over 14 days [10]. The only pediatric randomized controlled trial assessing steroid use for Bell’s palsy employed methylprednisolone at 1 mg/kg/day for 10 days, followed by a gradual taper over three to five days [11].

In Saudi Arabia, there remains a notable lack of research on Bell’s palsy in children, particularly concerning detailed demographics, the duration of illness, associated symptoms, complications, and recurrence rates. Addressing this gap is crucial for enhancing understanding and guiding management of the condition in this population. Therefore, this study aimed to identify the detailed demographics and assess the duration of illness, associated symptoms, complications, and recurrence rate of children diagnosed with Bell’s palsy at KAMC. In addition, we attempted to evaluate different treatments and compare prognostic factors.

## Materials and methods

Study design, area, settings, sample size, and sampling technique

A retrospective study was conducted on children aged 0 to 14 years who had been diagnosed with motor facial nerve palsy. The study included only Saudi patients, as it was performed at a military hospital that exclusively accepted Saudi nationals. Patients with other systemic diseases, including malignancies, hematological disorders, neurogenetic conditions, diabetes, or hypertension, were excluded. The research took place in Riyadh, Saudi Arabia, at King Abdullah Specialist Children’s Hospital (KASCH), a part of KAMC. The study focused on patients who had Bell’s palsy and were admitted through emergency room visits and clinics.

The total number of available patients was approximately 300. It was assumed that 50% of the sample would be male and the other 50% would be female. With a 95% confidence interval and an expected margin of error of 5%, the estimated sample size of 350 was calculated using Raosoft (Raosoft Inc., Seattle, WA). A non-probability consecutive sampling technique was employed. To minimize selection bias, all patients who met the inclusion criteria and received a final diagnosis of Bell’s palsy between 1997 and 2023 were included. Due to the limited number of pediatric patients with Bell’s palsy, all eligible patients were ultimately included in the study.

Data collection process

Data were collected by reviewing patients’ charts using a pre-designed data collection sheet for pediatric patients diagnosed with Bell’s palsy at KAMC. Patients’ electronic records were reviewed after obtaining the necessary authorization from the hospital’s medical record department. Detailed demographics, duration of illness, associated symptoms, complications, and recurrence rates were extracted.

The data were gathered from electronic medical records and entered into a standardized data collection sheet under the supervision of consultants. The chart review documented six key items: 1. Duration of illness and recovery (e.g., six months or three weeks) [6]; 2. Associated symptoms (e.g., jaw pain, drooling, headache) [12]; 3. Complications (e.g., irreversible damage to the facial nerve, abnormal nerve fiber regrowth) [12]; 4. Grouping variables: age, gender, date of admission

Data analysis

The data were entered and analyzed using the SAS statistical package, version 9.4 (SAS Inc., Cary, NC). Numerical variables (such as age and duration) were presented as means, medians, and standard deviations, while categorical variables (such as gender) were expressed as frequencies and percentages. Fisher’s exact test was used to determine associations between categorical variables, such as the presence of complications and recurrence. The chi-square test was utilized to assess associations between facial weakness and facial deviation. A p-value of less than 0.05 was considered statistically significant.

## Results

The study included 301 patients, with slightly over half identified as female (approximately 53%) and nearly half as male (around 47%). On average, the children were about 8.6 years old, and the mean length of stay in the hospital was just under two days (Tables 1, 2). 

**Table 1:** Profile of study participants (N=301)

Gender	Number	Percentage
Female	159	52.82%
Male	142	47.18%

**Table 2:** Mean age and length of hospital stay among participants

	Mean	STD
Age (years)	8.62	+-3.85
Length of stay (days)	1.92	+-5.55

Facial-related symptoms varied widely. Roughly two-thirds (about 68%) displayed some form of facial, mouth, or nasal angle deviation. Around 37% of the patients experienced facial weakness, while more than half did not. Inability to close the eye affected nearly 65% of patients. Facial numbness and headache were relatively rare, each occurring in only about 4% of the sample. Similarly, facial tenderness appeared in about 1% of cases. Tear production issues were present in roughly 7%. Additional symptoms, such as an inability to raise the eyebrow and the absence of forehead wrinkles, were less common, seen in about 8% and 14% of patients, respectively. Synkinesis, abnormal taste, and nasolabial fold abnormalities were even less frequent, each recorded in a small minority of patients (Table 3).

**Table 3:** Associated symptoms of Bell's palsy URTI: upper respiratory tract infection

Symptoms	Present	Not present
Facial/mouth/nasal angle deviation	206 (68.44%)	95 (31.56%)
Inability to close the eye	195(64.78%)	106 (35.22%)
Facial weakness	112 (37.21%)	189 (62.79%)
Absent forehead wrinkles	42 (13.95%)	259 (86.35%)
Facial/eye/ear/neck pain	28 (9.33%)	273 (90.67%)
Inability to elevate the eyebrow	25 (8.31%)	276 (91.69%)
Tearing of the eye	20 (6.64%)	281 (93.36%)
Headache	12(3.99%)	289 (96.01%)
Facial numbness	13(4.32%)	288 (95.68%)
Common cold/URTI	5.65 (17%)	284 (94.35%)
Abnormal tasting	8 (2.66%)	293 (97.34 %)
Absence of the nasolabial fold	32 (10.63%)	269 (89.37 %)
Synkinesis	4 (1.33%)	297 (98.67 %)
Facial tenderness	3(1.00%)	298 (99.00%)

In terms of complications, the overwhelming majority, approximately 94%, experienced no lasting problems. A small fraction (less than 1%) did not fully recover, and about 4% had persistent abnormal facial sensations. Vision disturbances and bilateral Bell’s palsy were each detected in less than 1% of patients (Table 4).

**Table 4:** Complications of Bell's palsy

Complications	Frequency	Percentages
No complication	281	93.98%
Persistent abnormal facial sensation	12	4.01%
Vision disturbance	2	0.67%
Bilateral Bell’s palsy	1	0.33%
Didn’t improve fully	2	0.67%
Prolonged case	1	0.33%

Treatment approaches varied. Oral prednisolone was widely used, administered to about 81% of patients. Eye care was also common, provided in about 72% of cases. Physiotherapy was offered to nearly half of the patients, while esomeprazole/omeprazole was given to roughly one-third. Acyclovir was rarely used, administered to only about 2% of patients (Table 5).

**Table 5:** Treatments for Bell's palsy

Treatments	Treatment was given	Treatment not given
Prednisolone	243 (80.73%)	58 (19.27%)
Eye care	216 (71.76%)	85 (28.24%)
Physiotherapy	146 (48.50%)	155 (51.50%)
Esomeprazole/omeprazole	95 (31.56%)	206 (68.44%)
Acyclovir	6 (1.99%)	295 (98.01%)

Regarding recurrence, the condition did not recur in about 80% of the cases. Approximately 14% experienced one recurrence, while a small fraction (around 6%) had two or more episodes (Table 6).

**Table 6:** Recurrence of Bell's palsy

Recurrence	Frequency	Percentage
0	242	80.40%
1	42	13.95%
2	8	2.66%
3	4	1.33%
4	3	1.00%
5	1	0.33%
9	1	0.33%

Inferential analysis

A statistically significant relationship emerged between facial weakness and the presence of facial deviation (p<0.001), indicating that patients with facial weakness were more likely to also have facial deviation (Table 7).

**Table 7:** Association between facial weakness and presence of facial deviation

Facial weakness	Facial deviation not present	Facial deviation present	Chi-square (χ²)	P-value (χ² test)
Facial weakness not present	46	143	12.6	0.0005
Facial weakness present	49	63		

Additionally, there was a significant association between experiencing complications and facing recurrence (p<0.001). This suggests that patients with any form of complication were at a higher risk of having Bell’s palsy recur (Table 8).

**Table 8:** Association between complications and recurrence of Bell’s palsy

Complications	Recurrence not present	Recurrence present	P-value (Fisher's exact test)
Had no complications	230	51	0.0002
Had complications	10	8	

## Discussion

This study aimed to address gaps in the understanding of Bell’s palsy among children at KAMC, including its clinical features, complications, recurrence rates, demographics, and the treatments employed. The findings showed a slightly higher prevalence among females (approximately 53%) and that most patients were older than eight years. Notably, nearly 68% of cases exhibited some degree of facial deviation. Encouragingly, about 94% of patients recovered without complications, and 80% did not experience recurrence. Treatment approaches frequently included oral steroids (prednisolone), given to around 80% of the patients.

Previous studies have also reported a female predominance [13,14], recording approximately 58% of cases in two separate cohorts. Similarly, while our mean patient age was around eight years, others have noted an average age closer to 11 [9,13]. The strong association found between facial weakness and facial deviation in our study suggests that these symptoms often co-occur. However, in contrast to a study reporting 100% eyelid muscle involvement [13], we recorded a lower frequency, possibly reflecting documentation bias or differing clinical assessments. Infections like upper respiratory tract infections were linked to Bell’s palsy in about 6% of our patients, whereas other studies found a higher association [13,15]. Variations in forehead and mouth muscle involvement between our cohort and others [13] further highlight potential differences in clinical presentation or the extent of facial nerve involvement.

Regarding pain, about 9% of our patients experienced facial, ear, eye, or neck pain. This contrasts with adult data [4,16], where pain was more common and often linked to incomplete recovery. Such differences underscore that in children, the facial nerve alone may not fully account for pain, hinting at possible involvement of the trigeminal nerve or other etiologies. Similarly, while about 4% of our patients experienced facial numbness, other studies [17] reported much higher frequencies in adults. Differences in tear production, headache prevalence, and abnormal taste perception between our findings and those reported by others [4,17] highlight the broad spectrum of clinical presentations and possibly differing underlying pathophysiology in pediatric cases.

Most children with Bell’s palsy enjoyed a benign course, with minimal complications. Persistent abnormal facial sensations in about 4% of cases might indicate more profound nerve damage. Only rare cases showed incomplete recovery, prolonged illness, or visual disturbances. In adults, visual issues occur more frequently [17], potentially due to lagophthalmos and dryness. Bilateral Bell’s palsy was exceedingly uncommon in our study, detected in only one case.

Treatment strategies remain somewhat controversial. Some children appear to recover well without any intervention, while others may benefit from timely steroid administration. Shih et al. [7] found no significant difference in recovery at three months with steroid use; however, overall outcomes still favored steroids. Youshani et al. [9] also noted the importance of early treatment. Although consensus is lacking, our data suggest that most clinicians favored a combination of steroids, eye care, and physiotherapy. Proton pump inhibitors were used prophylactically against steroid-induced peptic ulcers, and eye care was standard for those with incomplete eyelid closure. Physiotherapy likely supports regaining muscle function and symmetry.

Recurrence was documented in about 20% of cases, varying in frequency among individuals. Cirpaciu et al. [18] observed a lower recurrence rate of about 12% in their cohort, indicating that our higher recurrence figure may reflect differences in population or clinical follow-up. Importantly, we found that complications increased the likelihood of recurrence, emphasizing the need for close monitoring of patients showing early complications.

This study had limitations. We could not analyze the duration of illness due to limited follow-up, and the small sample size prevented us from excluding patients with systemic diseases that could influence outcomes. Despite these constraints, the findings contribute valuable insights into the clinical profile, management, and prognosis of pediatric Bell’s palsy and underscore the complexity of its presentation and the need for individualized treatment strategies.

## Conclusions

Bell’s palsy in children generally presents with a favorable prognosis, as the majority regain full function without complications. Females appear to be more commonly affected, and the average patient age ranges from eight to 12 years. Prominent symptoms include facial deviation and difficulty closing the eye. Although oral steroids are frequently administered, their clinical benefit remains uncertain and may offer only a minimal advantage. Recurrence remains a recognized feature of the condition, and multiple episodes may develop in individuals who are prone to repeated involvement. Despite this, established prognostic factors for recurrence are lacking. Additional research is necessary to determine the most effective treatments and to identify factors that may predict recurrence or complications.
